# Associations Between Duration of Lactation and Maternal Risk of Obesity‐Related Cancers—A Historical Birth Cohort Study

**DOI:** 10.1111/mcn.70240

**Published:** 2026-09-23

**Authors:** Sophie H. Christensen, Dorthe C. Pedersen, Julie Aarestrup, Kathleen M. Rasmussen, Jennifer L. Baker, Lise G. Bjerregaard

**Affiliations:** ^1^ Center for Clinical Research and Prevention Copenhagen University Hospital – Bispebjerg and Frederiksberg Hospital Copenhagen Denmark; ^2^ Department of Nutrition, Exercise and Sports University of Copenhagen Frederiksberg Denmark; ^3^ Diet, Cancer and Health Danish Cancer Institute Copenhagen Denmark; ^4^ Division of Nutritional Sciences Cornell University Ithaca New York USA

**Keywords:** breast cancer, cancer, endometrial cancer, lactation, obesity, ovarian cancer

## Abstract

Lactation may protect mothers against certain cancers, such as breast‐ and obesity‐related cancers (ORC). However, the evidence is limited and has methodological shortcomings. Therefore, we investigated the associations between lactation durations and the maternal risk of ORC as well as endometrial, ovarian, and postmenopausal breast cancer individually. We included 6880 mothers from the Copenhagen Perinatal Cohort giving birth between 1959 and 1961. Information on duration of predominant and any lactation was collected 1 year postpartum. We used the International Agency for Research on Cancer definition of ORC, including 13 cancers. Cancer diagnoses were obtained from The Danish Cancer Registry (*n*_ORC_ = 1300, *n*_endometrial_ = 97, *n*_ovarian_ = 89, *n*_postmenopausal breast_ = 591) during 1968‐2022. Hazard ratios (HR) and 95% confidence intervals (CI) for the cancer risk were estimated using Cox regressions adjusted for demographic, metabolic, and reproductive factors. Duration of any lactation was not associated with the risk of either ORC (HR = 1.00 [95% CI: 0.96–1.03] per month), or endometrial (0.95 [0.84–1.08] per month), ovarian (0.94 [0.82–1.09] per month) or postmenopausal breast (0.98 [0.94–1.03] per month) cancer individually. When we investigated the duration of predominant lactation, we found similar results. In this historical birth cohort study with 60 years of follow‐up, lactation was not associated with a clinically relevant reduction in maternal risk of ORC or of endometrial, ovarian or postmenopausal breast cancer individually. Prospective studies with longer durations and varying intensities of lactations are needed to elucidate potential associations more completely. Importantly, the lack of any directional findings supports leaving breastfeeding recommendations in place.

## Summary

- We examined associations of lactation and maternal risk of obesity‐related cancers, adjusting for potential confounders.
- Duration of lactation was not significantly associated with the risk of obesity‐related cancer.
- The results support leaving current breastfeeding recommendations in place.

## 1 Introduction

Each year, cancer affects more than 20 million persons and causes 9.7 million deaths worldwide (Cancer Today Internet 2025). Obesity is a major risk factor and has been linked with more than 13 forms of cancer as well as estimated to be accountable for 4%–8% of all cancer cases (Lauby‐Secretan et al. 2016; Pati et al. 2023; Sung et al. 2019; The World Health Organization 2025). The mechanisms explaining the association between obesity and subsequent cancer include activation of inflammatory processes in the adipose tissue, such as secretion of adipokines and cytokines (Lauby‐Secretan et al. 2016).

Among females, postpartum weight retention (PPWR) after a pregnancy may increase the risk of overweight and obesity (Linné et al. 2004). This can cause hormonal and metabolic changes, such as insulin resistance and hyperlipidaemia (Lauby‐Secretan et al. 2016; Louie et al. 2013), which may contribute to an increased risk of developing obesity‐related cancer (ORC) (Lauby‐Secretan et al. 2016; Hanahan and Weinberg 2011). As lactation reduces PPWR through mobilization of the adipose tissue (Baker et al. 2008), it may lower the risk of obesity and thereby potentially the risk of ORC (Stuebe and Rich‐Edwards 2009; Tahir et al. 2019; Foster et al. 2023). According to the “lactation reset hypothesis,” the hormonal and metabolic changes during pregnancy may reset more rapidly and more completely among women who lactate compared to those who do not (Stuebe and Rich‐Edwards 2009). Yet, studies investigating the potential link between lactation and the risk of ORC are missing at present.

The most prevalent ORC among females is postmenopausal breast cancer (BC) (World Cancer Research Fund 2025). A large meta‐analyses provided evidence for a protective influence of lactation on maternal pre‐ and postmenopausal BC, with up to a 4.3% risk reduction per 12 months of lactation (Collaborative Group on Hormonal Factors in Breast Cancer 2002). Furthermore, some evidence suggests that lactation may reduce the risk of ovarian and endometrial cancer (Collaborative Group on Hormonal Factors in Breast Cancer 2002; The World Cancer Research Fund 2020; World Cancer Research Fund. The Third Expert Report 2024; Wang et al. 2015; Babic et al. 2020; Jordan et al. 2017, 2012). Nonetheless, an important limitation of these studies is the long recall time of lactation information, especially in case‐control studies conducted in later adulthood, which potentially causes recall bias (Babic et al. 2020; Jordan et al. 2012). Moreover, information on important confounding factors, such as pre‐pregnancy body mass index (PPBMI) and socioeconomic position, is often lacking, thus uncertainty about the findings remains. With the prevalence of both obesity and cancer increasing among females of the reproductive age (Schon et al. 2024; Devlieger et al. 2016) and with obesity as an important risk factor for developing cancer (Sung et al. 2019; The World Health Organization 2025), it is important to investigate lactation as a potentially modifiable factor in relation to cancer prevention among females giving birth.

The objective of the present study was to investigate whether the duration of lactation is associated with the maternal risk of developing ORC, and endometrial, ovarian and postmenopausal breast cancer individually, while accounting for relevant confounding factors. We hypothesized that longer durations of lactation would be associated with a reduced risk of these cancer forms.

## 2 Materials and Methods

### 2.1 Data Sources

#### 2.1.1 The Copenhagen Perinatal Cohort

The Copenhagen Perinatal Cohort (CPC) includes women who gave birth in September 1959 to December 1961 at the National University Hospital in Copenhagen, Denmark (Zachau‐Christiansen and Ross 1975; Villumsen 1970; Christensen et al. 2025; Bjerregaard et al. 2019). The women were admitted based on the area of residence or if the women were expected to undergo a complicated delivery. However, most women were residents of Copenhagen and did not have complicated pregnancies. Mothers participated in an antenatal visit, an interview a few days postpartum, and a 1‐year follow‐up examination at which social, general medical and obstetric information was obtained.

#### 2.1.2 Lactation and Covariates

The durations of predominant and any breastfeeding (in days, weeks, and/or months) were assessed at the infant's 1‐y examination by a physician who recorded when the mother partly discontinued feeding the infant breast milk (defining the duration of predominant lactation) and when breastfeeding ceased (defining duration of any lactation). Information on maternal biological, metabolic, reproductive, and demographic factors was collected at birth and at the 1‐year follow‐up visit.

We considered the following as potential confounding factors: socioeconomic position (low, medium, or high), maternal PPBMI (kg/m^2^), maternal age (y), parity (birth order), maternal smoking during the third trimester (none, < 3, 3‐10 or > 10 cigarettes per day), infant sex (male/female) and diabetes during pregnancy (yes/no). These factors were chosen a priori using existing evidence and the use of directed acyclic graphs (DAGs) (Textor et al. 2016) (Supplementary Figure 1). Confounding factors were considered relevant for all outcomes examined.

Additional information, such as vital status and number of live births, was retrieved through linkage to the Danish Civil Registration System using the personal identification number, which has been issued to every Danish citizen since April 2, 1968 (Pedersen 2011).

#### 2.1.3 Case Ascertainment

Cancer diagnoses were obtained through linkage with the Danish Cancer Registry, which contains information on incident cancer diagnoses since 1943 (Gjerstorff 2011). In this study, diagnoses were defined according to the International Classification of Diseases (ICD) classification system version 7 until December 31, 1977, and version 10 from January 1, 1978, and onwards. Due to a recoding project, all cancers diagnosed between 1978 and 2004 were recoded to ICD‐version 10 (Gjerstorff 2011).

According to the International Agency for Research on Cancer, the 13 ORCs include colorectal, endometrial, gallbladder, gastric cardia, kidney, liver, meningioma, multiple myeloma, oesophageal, ovarian, pancreas, postmenopausal breast and thyroid cancer (Lauby‐Secretan et al. 2016) (Supplementary Table 1). Additionally, we analysed the female cancers— endometrial, ovarian and postmenopausal BC (defined as a BC diagnosis > 55 y of age, as most women have entered menopause at this age (Collaborative Group on Hormonal Factors in Breast Cancer 2019)—as separate outcomes.

### 2.2 Analytical Sample

**Figure 1:**
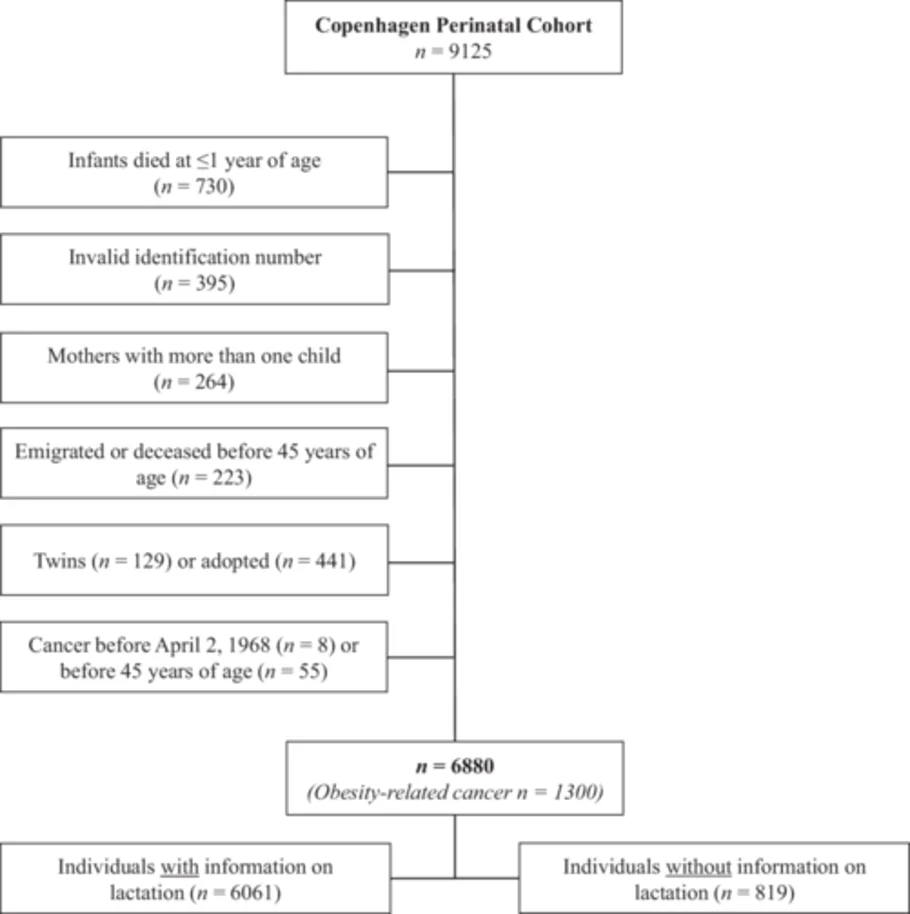
Flowchart of included individuals. A total of 6880 females were included in the present analysis of which 6061 had complete data and 819 were included with imputed lactation data. ORC = obesity‐related cancer.

We chose to include women from age 45 y to ensure that reproductive cycles had largely ended at the start of follow‐up, minimizing the potential influence of subsequent pregnancy and lactation cycles on the exposure‐outcome relationship. Mother–infant dyads were excluded if (1) the infant did not survive infancy (*n* = 730), (2) the mother had an invalid personal identification number (*n* = 395), 3) the mother had several children in the CPC (*n* = 264) in which case she was included only once with the first‐born child, (4) the mother emigrated or died before April 2, 1968 (*n* = 223), (5) the mother gave birth to twins (*n* = 129), the child was adopted (*n* = 441), or (6) the mother was diagnosed with cancer before April 2, 1968 (*n* = 8) or before 45 y of age (*n* = 55) (Figure 1). Follow‐up started at age 45 y and ended on the date of the first cancer diagnosis of interest, death, emigration, loss to follow‐up, or on December 31, 2022; whichever came first. In analyses on endometrial or ovarian cancer, women who had a hysterectomy or an oophorectomy before the age of 45 y (start of follow‐up) (*n* = 179) were excluded, and we censored women who had a hysterectomy or an oophorectomy after the age of 45 y on the date of these procedures.

### 2.3 Statistical Analyses

Descriptive data are presented overall and by lactation categories. Data are presented as median and interquartile range (IQR) for continuous variables or as counts (percentage, %) for categorical variables.

Cox proportional hazard regression analyses were performed to investigate whether longer durations of lactation were associated with a lower maternal risk of developing ORC and endometrial, ovarian and/or postmenopausal breast cancer individually. Age was used as the underlying time scale. Exposures included the duration of predominant and any lactation (months), respectively, and categories of any lactation at ≤ 0.5 months (ref.), > 0.5–1, > 1–2, > 2–4, > 4 months. Associations between lactation duration and cancer outcomes of interest were estimated without and with adjustment for demographic, metabolic, and reproductive factors e.g., socioeconomic position, PPBMI, maternal age, parity, maternal smoking in the third trimester and infant sex. Although diabetes during pregnancy was considered a possible confounding factor according to our DAG (Supporting Information S1: Figure 1), the low number of diagnoses (< 1%) precluded its inclusion and any sensitivity analyses. The proportions of missing data from the relevant confounding factors were 17% for socioeconomic position, 13% for the duration of predominant lactation, 10% for PPBMI, and 1% for smoking. We imputed missing information on either duration of predominant lactation, any lactation or any of the confounding factors using chained equations with 40 imputations based on all covariates, lactation variables, and the outcomes.

The proportional hazard assumption underlying the Cox model was tested using categories of age at risk (divided by the median age) using likelihood ratio tests. No violations across age were detected. Results are presented as hazard ratios (HR) with 95% confidence intervals (CI). All statistical analyses were carried out using Stata statistical software version 18.0 (StataCorp LLC, 2025, College Station, TX) and *mi impute* for multiple imputation of missing data.

#### 2.3.1 Ethics Statement

The study was approved by Danish Data Protection Agency (P‐2019‐832). According to Danish law, ethical approval is not required for purely register‐based studies.

## 3 Results

The final analytic sample size included 6880 females (75.4%) (Figure 1). Mothers had a median age at first birth of 23 (IQR: 20; 28) y, and a PPBMI of 21.4 (IQR: 19.8; 23.1) kg/m^2^ (Table 1). Around half of the mothers were primiparous, and they had a median duration of any lactation of 2.0 (IQR: 0.7; 4.0) months of which 1.4 (IQR: 0.5; 3.0) months was predominant lactation. Mothers lactating for ≤ 0.5 months were more likely to have had overweight and obesity prior to pregnancy and more often gave birth prematurely compared to the other groups (Table 1). In contrast, mothers who lactated for > 4 months were more likely to have a high socioeconomic position and to be non‐smokers (Table 1).

**Table 1:** Maternal and infant characteristics of the study population.

		Lactation categories				
Maternal characteristics	Total^a^ (*n* = 6880)	≤ 0.5 months (*n* = 1374)	> 0.5‐1 months (*n* = 888)	> 1‐2 months (*n* = 1191)	> 2‐4 months (*n* = 1349)	> 4 months (*n* = 1259)
Socio‐economic position^b^						
Low	1323 (19.2)	359 (26.1)	232 (26.1)	265 (22.3)	255 (18.9)	157 (12.5)
Medium	2424 (35.2)	536 (39.0)	390 (43.9)	480 (40.3)	532 (39.4)	425 (33.8)
High	1975 (28.7)	305 (22.2)	198 (22.3)	342 (28.7)	465 (34.5)	613 (48.7)
* Missing*	1158 (16.8)	174 (12.7)	68 (7.7)	104 (8.7)	97 (7.2)	64 (5.1)
Maternal age (y)	23 (20, 28)	23 (20, 28)	22 (19, 26)	23 (20, 27)	25 (21, 30)	23 (20, 28)
Maternal PPBMI (kg/m^2^)	21.4 (19.8, 23.1)	21.6 (19.9, 23.7)	21.4 (19.7, 23.1)	21.2 (19.7, 22.9)	21.2 (19.8, 22.9)	21.5 (19.8, 23.0)
* Missing*	706 (10.3)	166 (12.1)	101 (11.4)	96 (8.1)	127 (9.4)	100 (7.9)
Categories of maternal PPBMI (kg/m^2^)						
< 18.5	477 (7.8)	122 (8.9)	77 (8.7)	106 (8.9)	106 (7.9)	66 (8.4)
≥ 18.5& < 25	4388 (72.0)	895 (65.1)	633 (71.3)	893 (75.0)	987 (73.2)	980 (77.8)
≥ 25& < 30	522 (8.5)	154 (11.2)	65 (7.3)	87 (7.3)	115 (8.5)	101 (8.0)
≥ 30	84 (1.4)	37 (2.7)	12 (1.4)	9 (0.8)	14 (1.0)	12 (1.0)
*Missing*	590 (10.3)	166 (12.1)	101 (11.4)	96 (8.1)	127 (9.4)	100 (7.9)
Smoking (cigarettes per day)						
None	3345 (48.6)	608 (44.2)	389 (43.8)	541 (45.4)	646 (47.9)	785 (62.4)
< 3	485 (7.0)	88 (6.4)	65 (7.3)	90 (7.6)	94 (7.0)	87 (6.9)
3–10	2019 (29.3)	413 (30.1)	270 (30.4)	361 (30.3)	432 (32.0)	290 (23.0)
> 10	934 (13.6)	241 (17.5)	155 (17.5)	186 (15.6)	160 (11.9)	79 (6.3)
* Missing*	97 (1.4)	24 (1.7)	9 (1.0)	13 (1.1)	17 (1.3)	18 (1.2)
Infant characteristics						
Infant sex (female)	3390 (49.3)	653 (47.5)	447 (50.3)	579 (48.6)	669 (49.6)	627 (49.8)
Birth order of current child^a^	1 (1,2)	1 (1,2)	1 (1,2)	1 (1,2)	1 (1,2)	1 (1,2)
First‐born	4446 (64.6)	857 (62.4)	578 (65.1)	808 (67.8)	883 (65.5)	787 (62.5)
Preterm birth (yes)^c^	988 (14.4)	291 (21.2)	109 (12.3)	141 (11.8)	184 (13.6)	148 (11.8)
* Missing*	1300 (18.9)	274 (19.9)	184 (20.7)	233 (19.6)	257 (19.1)	202 (16.0)
Birth weight (g)	3.2 (2.9, 3.6)	3.1 (2.6, 3.5)	3.2 (3.0, 3.6)	3.2 (3.0, 3.6)	3.3 (3.0, 3.6)	3.4 (3.0, 3.7)
* Missing*	124 (1.8)	26 (1.9)	13 (1.5)	18 (1.5)	28 (2.1)	19 (1.5)
Duration of predominant lactation (months)	1.4 (0.5, 3.0)	0.2 (0.2, 0.2)	0.8 (0.7, 1.0)	1.5 (1.0, 2.0)	3.0 (2.0, 3.0)	4.5 (3.0, 6.0)
*Missing*	880 (12.8)	—	—	—	—	—

*Note:* Data are presented as proportions (%) or medians (interquartile range). PPBMI = pre‐pregnancy body mass index.

^a^ The entire cohort comprises *n* = 6880 females of which only *n* = 6061 had complete lactation data and were divided into categories of lactation duration. Thus, the total horizontally for certain rows is *n* = 6061.

^b^ Points for socioeconomic position were given according to occupation of the primary income‐earner of the household, the way in which the primary income‐earner earn his/her wages, the training of the primary income‐earner and the character of the living accommodation (0–5 points per indicator). Low = 0–6 points, medium = 7–10 points, high = 11–20 points.

^c^ Defined as < 38 weeks of gestational age.

Of the 6880 mothers, 1300 (18.9%) were diagnosed with an ORC during follow‐up. Among these, 97 (7.5%) were diagnosed with endometrial, 89 (6.8%) with ovarian, and 591 (45.5%) with postmenopausal BC (Table 2). In all lactation groups, the most common cancer form was postmenopausal BC (Table 2), and the mothers had a mean follow‐up time of 29 y (202,186 total person‐years).

**Table 2:** Cancer cases overall and by lactation categories.

Cancer form	Lactation categories					
	Total^a^ (*n* = 6880)	≤ 0.5 months (*n* = 1374)	> 0.5‐1 months (*n* = 888)	> 1‐2 months (*n* = 1191)	> 2‐4 months (*n* = 1349)	> 4 months (*n* = 1259)
Obesity‐related^b^	1300 (18.9)	230 (16.7)	158 (17.8)	241 (20.2)	252 (18.7)	287 (22.8)
Endometrial	97 (1.4)	14 (1.0)	10 (1.1)	28 (2.4)	16 (1.2)	21 (1.7)
Ovarian	89 (1.3)	19 (1.4)	10 (1.1)	18 (1.5)	17 (1.3)	18 (1.4)
Postmenopausal breast	591 (8.6)	103 (7.5)	75 (8.4)	113 (9.5)	120 (8.9)	127 (10.0)

*Note:* Data are presented as proportions (%).

^a^ The entire cohort comprises *n* = 6800 individuals of which only *n* = 6061 had complete lactation data and were divided into categories of lactation duration. Thus, the total n for each cancer form when adding the cancer cases from each lactation category does not add up to the total cases.

^b^ Obesity‐related cancer included colorectal, endometrial, gallbladder, gastric cardia, kidney, liver, meningioma, multiple myeloma, oesophageal, ovarian, pancreas and thyroid cancer.

In the adjusted models using lactation duration as a continuous variable, durations of predominant (HR = 1.03 [95% CI = 0.98;1.08] per month) or any (HR = 1.00 [95% CI = 0.97;1.03] per month) lactation were not associated with the risk of ORC (Table 3). Similarly, duration of predominant or any lactation was not associated with either endometrial, ovarian, or postmenopausal breast cancer (Table 3).

**Table 3:** Associations between the duration of predominant and any lactation and cancer risk^a^.

Cancer form	Cases^a^	Duration of predominant lactation (months), *n* = 6880			
		Unadjusted		Adjusted^b^	
		HR	95% CI	HR	95% CI
Obesity‐related^c^	1300 (18.9)	1.03	[0.98; 1.08]	1.02	[0.97; 1.08]
Endometrial	97 (1.4)	1.03	[0.85; 1.26]	1.03	[0.84; 1.27]
Ovarian	89 (1.3)	1.08	[0.87; 1.33]	1.08	[0.87; 1.33]
Postmenopausal breast	591 (8.6)	1.03	[0.95; 1.11]	1.02	[0.95; 1.10]

		Duration of any lactation (months), *n* = 6880			
		Unadjusted		Adjusted^b^	
		HR	95% CI	HR	95% CI
Obesity‐related^c^	1300 (18.9)	1.00	[0.97; 1.03]	1.00	[0.96; 1.03]
Endometrial	97 (1.4)	0.97	[0.86; 1.11]	0.94	[0.82; 1.08]
Ovarian	89 (1.3)	0.95	[0.83; 1.10]	0.94	[0.82; 1.09]
Postmenopausal breast	591 (8.6)	0.99	[0.94; 1.04]	0.98	[0.94; 1.03]

^a^ Hazard ratios (HR) and 95% confidence intervals (CI) were estimated using Cox proportional hazards regression analyses.

^b^ Model adjusted for socio‐economic position, pre‐pregnancy body mass index, age at first child, parity, smoking in the third trimester and infant sex.

^c^ Obesity‐related cancer included colorectal, endometrial, gall bladder, gastric cardia, kidney, liver, meningioma, multiple myeloma, oesophageal, ovarian, pancreas and thyroid cancer.

**Figure 2:**
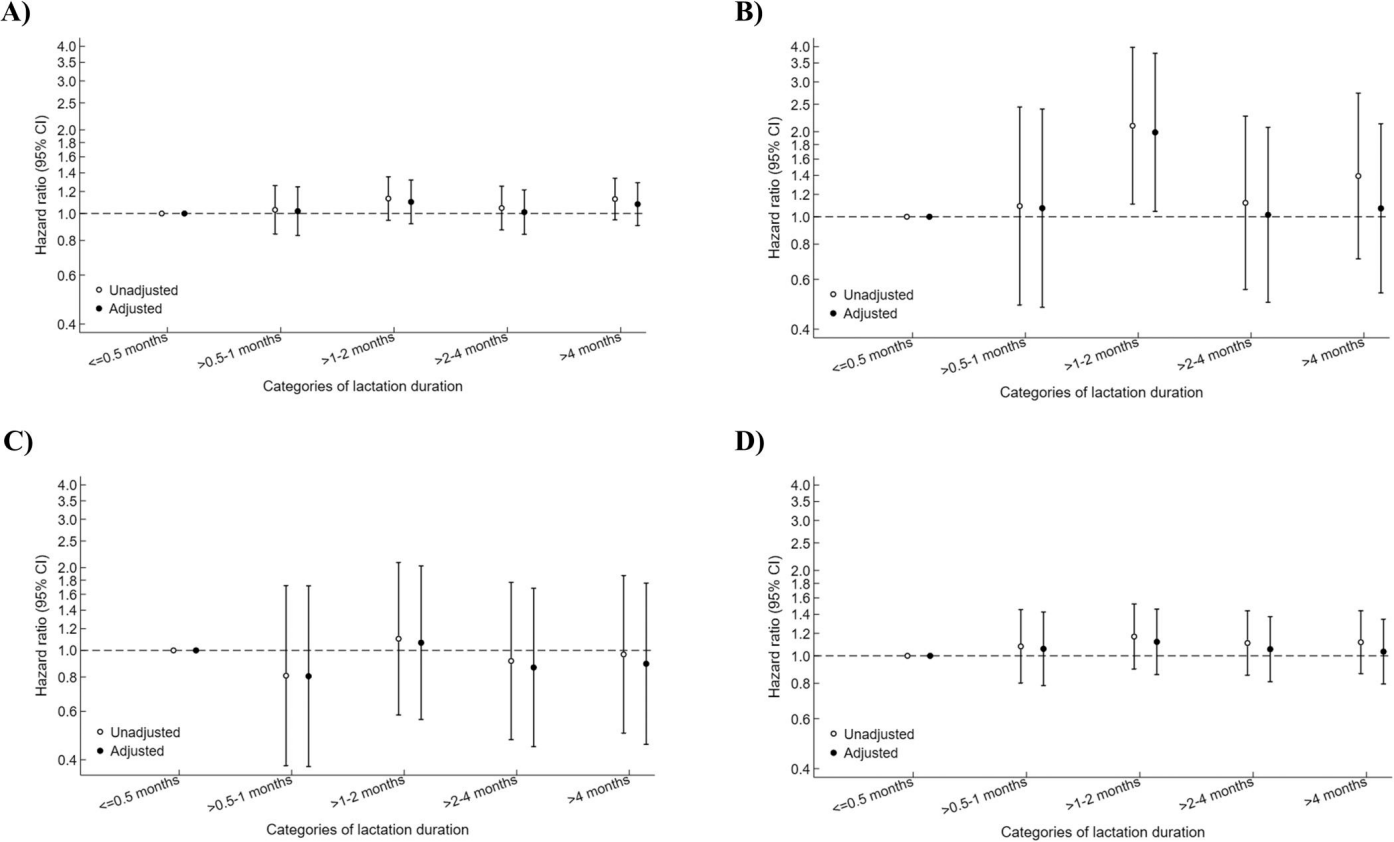
Plots of hazard ratio of cancer risk by categories of lactation duration. (A) Obesity‐related cancer, (B) Endometrial cancer, (C) Ovarian cancer, (D) Postmenopausal breast cancer. The lactation category “≤ 0.5 months” was used as the reference. Adjusted models included socio‐economic position, pre‐pregnancy body mass index, maternal age, parity (birth order), smoking in the third trimester and infant sex as confounding factors. CI = confidence interval.

In both unadjusted and adjusted analyses using the duration of any lactation as a categorical variable, categories of any lactation were not associated with the risks of ORC, endometrial, ovarian and postmenopausal BC (Figure 2 and Supplementary Table 2). One exception was that in both the unadjusted and adjusted models, compared to lactation for ≤ 0.5 months, any lactation for > 1–2 months was associated with a HR of 2.10 (1.11–3.98) and 1.99 (1.04–3.79), respectively, for the association with endometrial cancer (Figure 2 and Supporting Information S1: Table 2).

## 4 Discussion

In the present study, we did not find that the duration of lactation was associated with the maternal risk of developing either ORC or endometrial, ovarian, or postmenopausal breast cancer individually. Additionally, our findings were similar for predominant and any lactation. Lactation for > 1–2 months, compared to ≤ 0.5 months, was associated with a marginally increased risk of endometrial cancer, yet confidence intervals were wide. Our results were consistent across unadjusted models and models adjusted for relevant confounding factors.

We did not identify other studies investigating the influence of lactation on the risk of ORC limiting comparison with findings from previous studies. As such, we can only speculate whether the lack of an association in our study reflects methodological constraints or a true non‐association. We hypothesized that lactation reduces the risk of ORC through a reduced PPWR (Baker et al. 2008) and thereby reduced overweight and obesity postpartum (Stuebe and Rich‐Edwards 2009; Tahir et al. 2019; Foster et al. 2023). One explanation for the lack of an association could be a low variance of overweight and obesity postpartum in our population, which may limit the ability to detect an association between duration of lactation and risk of ORC. Yet, we do not have information on weight status after pregnancy. However, we observed limited variation in PPBMI across the lactation groups, with only 9% of females having either overweight or obesity, a proportion comparable to the prevalence of obesity in Copenhagen in the 1960s (Heitmann et al. 2004). Thus, we speculate that we would have a similarly low variance in the prevalence of overweight and obesity postpartum. Distinguishing the impact of lactation from that of postpartum overweight and obesity on the risk of ORC is important in future studies in populations with a higher prevalence of overweight and obesity.

Our findings of a marginal increase in the risk of endometrial cancer for females lactating for > 1–2 months, compared to ≤ 0.5 months, was not consistent across lactation groups, i.e., no duration‐response association was apparent. Although the results were significant in both the unadjusted and adjusted analyses, the estimates had wide CIs and should be interpreted cautiously. Our findings are in contrast to those of two large meta‐analyses, both of which found that mothers who lactated for longer periods had a 26% (Zhan et al. 2015) and 29% (Wang et al. 2015) reduced risk of endometrial cancer, respectively, compared to those with shorter durations or no lactation. Importantly, ≥ 24 months of any lactation was typically used as the category of “longer durations,” while mothers with the longest duration in our study lactated for ≥ 4 months with a median duration of any lactation of 7.0 months. In dose‐response analyses, both meta‐analyses found a significant protective effect of longer lactation duration on endometrial cancer risk, with a risk reduction of up to 2% per month of lactation (Wang et al. 2015; Zhan et al. 2015)—an association we were unable to replicate.

We were unable to detect any association between duration of lactation and the maternal risk of either postmenopausal BC or ovarian cancer. According to current evidence, lactation may protect against these cancer forms (The World Cancer Research Fund 2020; World Cancer Research Fund. The Third Expert Report 2024; Breast cancer and breastfeeding 2002). This conclusion is based on meta‐analyses from 2017 including data from 13 (*n* = 11,610 BC cases) and three (*n* = 817 ovarian cancer cases) cohort studies, respectively (World Cancer Research Fund. The Third Expert Report 2024). For BC, the authors found a 2% risk reduction per 5 months of lactation. However, in a separate dose‐response meta‐analysis, the results were not statistically significant for postmenopausal BC, in accord with our findings. Further, another large meta‐analysis from 2002 including five cohort and 38 case‐control studies investigating pre‐ and postmenopausal BC (*n* = 50,302 cases) estimated a 4.3% risk reduction per 12 months of lactation after adjustment for important confounding factors such as ethnic origin, education, family history of BC, parity and body mass index (Breast cancer and breastfeeding 2002).

Several factors may explain the inconsistency between others' findings of a protective influence of lactation on BC and our results showing null findings. First, the comparability of studies is often limited by differences in how lactation is reported, e.g., ever/never lactated versus duration of lactation and whether data were collected retro‐ or prospectively. Studies also differ in whether they include lactation intensity, i.e., predominant versus any lactation. Second, the recall time of lactation data in case‐control studies varied between 6 and 20 y (The World Cancer Research Fund 2020), and longer recall periods increase the risk of recall bias in these studies (Natland et al. 2012). The recall time in this cohort of 1 year limits the risk of recall bias, although we cannot preclude this. Third, as lactation may improve the hormonal and metabolic milieu postpartum compared to during pregnancy, the number of pregnancies and subsequent lactational cycles may affect the cancer risk. We were only able to include one individual lactation cycle from each mother, and it is possible that lifetime history of lactation may have a stronger influence on cancer risk than individual lactation cycles. Lifetime duration of lactation was used in the meta‐analysis from 2002, where they found a 4.3% decreased risk of BC per 12 months of lifetime duration of lactation (Breast cancer and breastfeeding 2002). Fourth, information on important confounding factors is not consistently available among current studies investigating lactation and BC risk (The World Cancer Research Fund 2020; Breast Cancer and breastfeeding 2002). As an example, PPBMI increases the risk of both early cessation of lactation (Hashemi‐Nazari et al. 2020) and the risk of a higher PPBMI in subsequent pregnancies (Marchewka‐Długońska et al. 2025; KETTERL et al. 2018). Thus, PPBMI possibly increases the risk of postmenopausal BC and ORC and should be accounted for when investigating the influence of lactation on these cancer forms. Additionally, cohort‐specific characteristics may further explain the differences between others and our findings. Finally, the meta‐analyses included both pre‐ and postmenopausal BC, which may differ in their pathophysiological response to pregnancy and lactation (Jindal et al. 2014; Gabrielson et al. 2018). In contrast, our study focused exclusively on postmenopausal BC, which may partly explain the discrepancies between our findings and those reported in previous meta‐analyses.

### 4.1 Strengths and Limitations

The main strengths of our study include the prospective study design with 60 years of follow‐up and the collection of lactation data shortly after it ceased, which enhances the validity of our lactation data. The thorough collection of important confounding factors, such as PPBMI, parity, age, and smoking minimize the risk of confounded results. However, it is uncertain whether our findings can be generalized to populations with a higher proportion of overweight and/or obesity and different reproductive behaviour. Lastly, the follow‐up in the Danish Cancer Registry is nearly complete, as the risk of non‐reported cases is low due to mandatory reporting. The internal validity of our findings is further enhanced by the fact that the diagnoses are morphologically verified. Moreover, our relatively large number of ORC cases (*n* = 1300) strengthens our statistical power for examining the association with lactation.

In addition, our study had some limitations. First, we had a low number of cases of endometrial and ovarian cancers, which reduced our statistical power and resulted in wide CIs for these cancers. This may also contribute to the diverging results between ours and others' findings, e.g., the meta‐analysis by the Collaborative Group on Hormonal Factors in Breast Cancer (Breast cancer and breastfeeding 2002). Second, we had only limited variation in lactation durations and lactation patterns. Our lactation data were collected between 1960 and 1962, when lactation durations were generally shorter than current durations, where Danish infants are fully breastfed for 3.5 months on average (The Danish Health Data Authority 2024). Only 21% of women reported any lactation for > 4 mo. However, this duration is within the range observed in the Copenhagen Municipality in 1958–62 (32% among infants born within marriage and 18% among those born outside of marriage) (Biering‐Sørensen et al. 1980). The limited variance in lactation patterns resulted in the durations of predominant and any lactation being relatively similar. This indicates that mothers ceased both predominant and any lactation almost simultaneously, which reduced our ability to fully explore the influence of lactation intensity on these outcomes. Third, the women gave birth at a time when maternal age at birth, lifetime reproductive history, BMI distributions, hormone exposure, and lifestyle factors differed from today. These factors may have affected their baseline cancer risk. Further studies are needed to investigate whether the findings are generalizable to mothers in other countries and more contemporary populations with longer durations of lactation. Finally, unmeasured confounding from cancer risk factors at baseline, such as dietary intake, physical activity, alcohol consumption, use of medication and family history of cancer, may have affected our results. Further, we did not account for cancer risk factors later in life such as health behaviour and metabolic conditions. However, these factors may potentially be mediators, but not confounders, and we did not aim to investigate mediation.

## 5 Conclusion

We found no associations between the duration of lactation and the risk of developing either ORC or endometrial, ovarian, or postmenopausal breast cancer individually in this prospective longitudinal cohort study. The results should be interpreted within the limitations of the relatively short durations of lactation. Prospective studies with longer durations and varying intensities of lactations and adjustment for confounding factors are needed to elucidate the potential associations more completely. Importantly, our findings of no beneficial or detrimental association between lactation duration and ORC do not diminish the well‐established health benefits of lactation for both mother and infant and support maintaining current breastfeeding recommendations.

## Author Contributions

SCH, LGB: designed the research. JLB, LGB: provided the databases. SCH: conducted the analyses. DCP, JA, JLB, KMR: contributed to the specification of the analyses. SCH, LGB: wrote the manuscript. SCH: had primary responsibility for the final content; and all authors: critically read and edited the manuscript and read approved the final manuscript.

## Conflicts of Interest

Unrelated to this work JLB reports consulting for Novo Nordisk with all fees paid to her institute. All other authors declare no conflicts of interest.

## Supporting information


Supporting File

## Data Availability

The data used in this study are based on a combination of data with personal identification numbers from the CPC, hosted by the Center for Clinical Research and Prevention, and data from national health registers. According to Danish law, this information cannot be publicly available. Access to the subset of data included in this study can be gained through submitting a project application to the corresponding author and pending approval by the data steering committee.
