# Supplementary material for: Associations Between Duration of Lactation and Maternal Risk of Obesity‐Related Cancers—A Historical Birth Cohort Study
Source: Matern Child Nutr. 2026 Sep 23;22(3):e70240. doi: 10.1111/mcn.70240 (PMC13599358; doi:10.1111/mcn.70240)
Supplement: Supplementary file 1 [file MCN-22-e70240-s001.docx]

# Supplementary Material

**Supplementary Figure 1. Directed Acyclic Graph of the association between duration of lactation and the maternal risk of developing cancer.** Black arrows represent unbiased pathways after correct adjustment for confounding factors. = exposure; = outcome; = adjusted confounding factors; = unadjusted confounding factor; = ancestors of exposure; = other variables.

| Supplementary Table 1. ICD-7 and ICD-10 codes used to define maternal cancers in the study sample of individuals from the Copenhagen Perinatal Cohort. | | |
| --- | --- | --- |
| Cancer Site | ICD-7 | ICD-10 |
| Colorectal | 153.0, 153.4-153.5, 253.0-253.4, 453.0-453.5, 453.8, 853.0-853.5, 753.0-753.5, 753.8, 154.0, 454.0, 754.0, 854.0 | C18.0-18.9,  C19.9, C20.9 |
| Endometrial | 172.0-172.2, 472.0, 872.0, 772.0, 775.0, 173.0, 473.0, 873.0, 174.0, 174.1, 474.0, 874.0, 774.0 | C54, C55 |
| Gallbladder* | 155.1, 155.3, 455.2, 455.3, 755.1, 755.3, 855.1, 855.3 | C23 |
| Gastric Cardia | 151.0-151.3, 451.0-451.3, 751.0-751.3, 851.0-851.3 | C16 |
| Kidney | 180.0-180.3, 480.0-480.1, 480.4, 880.0, 880.2, 780.0 | C64 |
| Liver | 155.0, 156.9, 455.0, 755.0, 855.0, 856.0 | C22 |
| Meningioma | 293.1, 493.2 | C70 |
| Multiple myeloma | 203.0-203.3 | C90 |
| Oesophageal | 150.0-150.2, 450.0, 750.0, 850.0 | C15 |
| Ovarian | 175.0-175.3, 375.0, 475.0, 875.0, | C56 |
| Pancreas | 157.0, 457.0, 757.0, 857.0 | C25 |
| Thyroid | 194.0, 494.0, 794.0, 894.0 | C73 |
| Postmenopausal breast cancer ** | 170.0-170.5, 470.0-470.5, 770.0-770.3, 870.0-870.2 | C50.0 – C50.9, D05.0 – D05.9 |
| *Cancer includes gall bladder and bile duct  **Postmenopausal breast cancer was defined as ≥55 y of age as most females have entered menopause at this age.  IARC = International Agency for Research on Cancer | | |

| Supplementary Table 2. Associations between categories of any lactation durations and cancer risk^1^. | | | | | | |
| --- | --- | --- | --- | --- | --- | --- |
|  |  | **Unadjusted** | |  | **Adjusted^2^** | |
| *Cancer* | | ***HR*** | ***95% CI*** |  | ***HR*** | ***95% CI*** |
| Obesity-related^3^  *(n=1300, 18.9 %)* | |  |  |  |  |  |
|  | >0.5-1 months | 1.03 | [0.84; 1.26] |  | 1.02 | [0.83; 1.25] |
|  | >1-2 months | 1.13 | [0.95; 1.36] |  | 1.10 | [0.92; 1.32] |
|  | >2-4 months | 1.05 | [0.87; 1.25] |  | 1.01 | [0.84; 1.22] |
|  | >4 months | 1.13 | [0.95; 1.34] |  | 1.08 | [0.90; 1.29] |
|  |  |  |  |  |  |  |
| Endometrial  *(n=97, 1.4 %)* | |  |  |  |  |  |
|  | >0.5-1 months | 1.09 | [0.49; 2.44] |  | 1.07 | [0.48; 2.41] |
|  | >1-2 months | **2.10** | **[1.11; 3.98]** |  | **1.99** | **[1.04; 3.79]** |
|  | >2-4 months | 1.12 | [0.55; 2.27] |  | 1.02 | [0.50; 2.07] |
|  | >4 months | 1.39 | [0.71; 2.74] |  | 1.07 | [0.54; 2.13] |
|  |  |  |  |  |  |  |
| Ovarian  *(n=89, 1.3 %)* | |  |  |  |  |  |
|  | >0.5-1 months | 0.79 | [0.37; 1.70] |  | 0.79 | [0.37; 1.71] |
|  | >1-2 months | 1.04 | [0.54; 1.97] |  | 1.00 | [0.52; 1.92] |
|  | >2-4 months | 0.87 | [0.45; 1.67] |  | 0.83 | [0.43; 1.59] |
|  | >4 months | 0.91 | [0.48; 1.74] |  | 0.84 | [0.43; 1.62] |
| Postmenopausal breast  *(n=591, 8.6 %)* | |  |  |  |  |  |
|  | >0.5-1 months | 1.08 | [0.80; 1.45] |  | 1.06 | [0.79; 1.43] |
|  | >1-2 months | 1.20 | [0.90; 1.52] |  | 1.12 | [0.86; 1.46] |
|  | >2-4 months | 1.11 | [0.85; 1.44] |  | 1.06 | [0.81; 1.37] |
|  | >4 months | 1.12 | [0.86; 1.44] |  | 1.03 | [0.80; 1.34] |
| ^1^Lactation was estimated as any lactation (reference group = lactation for ≤0.5 months) and hazard ratios (HR) and 95% confidence intervals (CI) were estimated using Cox proportional hazards regression analyses.  ^2^Model adjusted for socio-economic position, pre-pregnancy body mass index, age at first child, parity, smoking in third trimester and infant sex.  ^3^Obesity-related cancer included colorectal, endometrial, gall bladder, gastric cardia, kidney, liver, meningioma, multiple myeloma, oesophageal, ovarian, pancreas and thyroid cancer.  Significant results (p-value <0.5) are indicated in bold. | | | | | | |
